# sstar: A Python Package for Detecting Archaic Introgression from Population Genetic Data with S*

**DOI:** 10.1093/molbev/msac212

**Published:** 2022-10-01

**Authors:** Xin Huang, Patricia Kruisz, Martin Kuhlwilm

**Affiliations:** Department of Evolutionary Anthropology, University of Vienna, Djerassiplatz 1, 1030 Vienna, Austria; Human Evolution and Archaeological Sciences (HEAS), University of Vienna, Djerassiplatz 1, 1030 Vienna, Austria; Department of Bio Data Science, Faculty of Engineering, University of Applied Sciences Wiener Neustadt, Biotech Campus Tulln, Konrad Lorenz-Straße 10, 3430 Tulln, Austria; Department of Evolutionary Anthropology, University of Vienna, Djerassiplatz 1, 1030 Vienna, Austria; Human Evolution and Archaeological Sciences (HEAS), University of Vienna, Djerassiplatz 1, 1030 Vienna, Austria

**Keywords:** introgression, archaic admixture, S*, Python

## Abstract

S* is a widely used statistic for detecting archaic admixture from population genetic data. Previous studies used freezing-archer to apply S*, which is only directly applicable to the specific case of Neanderthal and Denisovan introgression in Papuans. Here, we implemented sstar for a more general purpose. Compared with several tools, including SPrime, SkovHMM, and ArchaicSeeker2.0, for detecting introgressed fragments with simulations, our results suggest that sstar is robust to differences in demographic models, including ghost introgression and two-source introgression. We believe sstar will be a useful tool for detecting introgressed fragments in various scenarios and in non-human species.

Admixture between populations is a topic of great interest (Fontsere et al. 2019), especially in hominins (Peter 2020). To detect archaic admixture from population genetic data, a statistic named S* was introduced to search for patterns of variation and linkage expected in the case of introgression (Plagnol and Wall 2006). This statistic has been applied in subsequent studies in modern humans (Wall et al. 2009; Huerta-Sanchez et al. 2014; Vernot and Akey 2014; Vernot et al. 2016; Xu et al. 2017; Jacobs et al. 2019), as well as other organisms (Cong et al. 2016; Kuhlwilm et al. 2019). Although the S* statistic is a powerful approach for detecting introgressed fragments without source genomes, there is no user-friendly and versatile package available. A previous implementation of S* is freezing-archer, which was specifically designed with human demographic models and used for detecting introgressed fragments from Neanderthals and Denisovans into Papuans (Vernot et al. 2016). Users must carefully read and understand the source codes of freezing-archer before manually changing the parameters inside the code. To improve the efficiency, robustness and reproducibility when using S* for detecting introgression, we implemented sstar.

The whole pipeline is illustrated in figure 1*A*. We define the population without introgressed fragments as the reference population, the population that received introgressed fragments as the target population, and the population that donated introgressed fragments as the source population (supplementary fig. S1, Supplementary Material online). We assume genotype data are diploid, biallelic and not missing in all the individuals of a dataset. We remove variants with derived alleles that are fixed in both the reference and target populations. Users can calculate S* for sliding windows across genomes by defining the window length and step size. To assess significance of S* scores, users can simulate data under a demographic model without introgression and build a generalized additive model (GAM) with different S* scores, quantiles of S*, numbers of mutations, and local recombination rates to predict the expected S* scores, as described previously (Vernot et al. 2016). If a genome from a potential source population is available, users can calculate the source match rate between an individual from the target population and an individual from the source population. If genomes from two different source populations are available, the origin of candidate introgressed fragments can be determined by comparing the source match rates with different source populations.

**Fig. 1.**
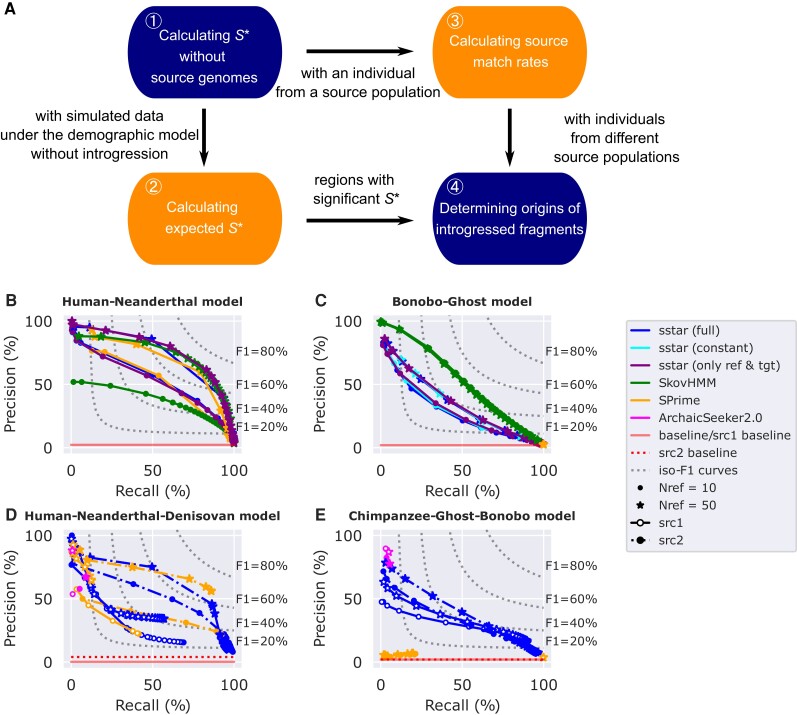
The sstar workflow and its performance in different demographic models and sample sizes with SPrime, SkovHMM, and ArchaicSeeker2.0. Different points represent precision and recall estimated with different cutoffs (supplementary tables S1–S6, Supplementary Material online). For ArchaicSeeker2.0, we only used the best results because this tool does not provide options to define candidate introgressed fragments with different cutoffs. Nref is the diploid sample size of the reference population (10 or 50). sstar (full) are results inferred with GAMs using simulated data from full demographic models without introgression (supplementary figs. S6–S9, Supplementary Material online). sstar (constant) are results inferred with GAMs using simulated data from constant effective population size models without introgression (supplementary figs. S10 and S11, Supplementary Material online). sstar (only ref and tgt) are results inferred with GAMs using simulated data from models with only the reference and target populations, these populations are also constant in size (supplementary figs. S12 and S13, Supplementary Material online). src1 represents the performance for identifying the introgressed fragments from the source population 1. src2 represents the performance for identifying the introgressed fragments from the source population 2. A baseline is the performance of a random classifier, where the precision is equal to the true proportion of the introgressed fragments. An *F*_1_ score is the harmonic mean of a given pair of precision and recall, dotted hyperbolic curves represent *F*_1_ isometrics. (*A*) The sstar workflow. (*B*) The precision-recall curves of sstar, SPrime, and SkovHMM for detecting introgression without source genomes under a Human-Neanderthal model(Gower et al. 2021, supplementary fig. S2 and table S7, Supplementary Material online). (*C*) The precision-recall curves of sstar, SPrime, and SkovHMM for detecting introgression without source genomes under a Bonobo-Ghost model(Kuhlwilm et al. 2019, supplementary fig. S3 and table S8, Supplementary Material online). (*D*) The precision-recall curves of sstar, SPrime, and ArchaicSeeker2.0 for detecting introgression with source genomes from two-source populations under a Human-Neanderthal-Denisovan model from stdpopsim(Adrion et al. 2020, supplementary fig. S4 and table S9, Supplementary Material online). The src1 population is the Neanderthal population. The src2 population is the Denisovan population. (*E*) The precision-recall curves of sstar, SPrime, and ArchaicSeeker2.0 for detecting introgression with source genomes from two-source populations under a hypothetical Chimpanzee-Ghost-Bonobo model(supplementary fig. S5 and table S10, Supplementary Material online) modified from Kuhlwilm et al. (2019). The src1 population is the Ghost population, and the src2 population is the Bonobo population, both introgressing into the Central Chimpanzee population.

We evaluated the performance of sstar with precision-recall curves because precision-recall curves may be more informative than receiver operating characteristic curves on imbalanced data sets (Saito and Rehmsmeier 2015). We simulated data with msprime 1.0 (Kelleher et al. 2016; Baumdicker et al. 2022) for different demographies and sample sizes. Two models tested ghost introgression: a Human-Neanderthal model (Gower et al. 2021) and a Bonobo-Ghost model (Kuhlwilm et al. 2019). Two further models tested two-source introgression: a Human-Neanderthal-Denisovan model (Malaspinas et al. 2016; Jacobs et al. 2019) and a Chimpanzee-Ghost-Bonobo model. For ghost introgression, we compared sstar with SPrime (Browning et al. 2018), another tool using an S*-like approach, and SkovHMM (Skov et al. 2018), a tool based on hidden Markov models (HMMs). In the Human-Neanderthal model, our results show that sstar and SPrime performed better than SkovHMM, when sample size was small (ten reference individuals, fig. 1*B*). In the Bonobo-Ghost model, SPrime performed poorly (fig. 1*C*), assigning the whole simulated sequence as a single introgressed fragment, while sstar and SkovHMM still detected introgressed fragments.

One key step in sstar is calculating the expected S* scores with simulated data from demographic models without introgression, requiring detailed knowledge on population history (supplementary figs. S6–S9, Supplementary Material online). Using approximate models (supplementary figs. S10–S13, Supplementary Material online), our results suggest that sstar still performed similarly to those using the full history (fig. 1*B*and*C*). For two-source introgression, we compared sstar with SPrime, and ArchaicSeeker2.0 (Yuan et al. 2021; Zhang et al. 2022), another HMM-based tool. Both sstar and SPrime performed better when identifying Denisovan fragments than identifying Neanderthal fragments (fig. 1*D*). This may be due to the Denisovan introgression event in Papuans being more recent and its admixture proportion being larger than for the Neanderthal introgression. More ancient events like in the Chimpanzee-Ghost-Bonobo model cannot be well determined by SPrime, while sstar still retained power (fig. 1*E*).

We conclude that sstar is robust for detecting introgressed fragments. Since no single tool could perform well in all scenarios, users should choose appropriate tools based on their data. We believe sstar will be useful in various scenarios, especially considering small samples, and non-human data sets.

## Supplementary Material

msac212_Supplementary_Data

## Data Availability

Source codes for sstar can be found in https://github.com/admixVIE/sstar (last accessed on August 17, 2022) and the manual can be found in https://sstar.readthedocs.io/en/latest/ (last accessed August 17, 2022). Codes for replicating the benchmarking can be found in https://github.com/admixVIE/sstar-analysis (last accessed August 17, 2022). Computational tools installed through conda can be found in https://github.com/admixVIE/sstar-analysis/blob/main/environment.yml (last accessed August 17, 2022). Tools listed below cannot be installed through conda but can be found in the websites from their authors: ArchaicSeeker2.0 (https://github.com/Shuhua-Group/ArchaicSeeker2.0, last accessed August 17, 2022), ms program ([Hudson 2002]; https://home.uchicago.edu/∼rhudson1/source/mksamples.html, last accessed August 17, 2022), SkovHMM (https://github.com/LauritsSkov/Introgression-detection, last accessed August 17, 2022), SPrime (https://github.com/browning-lab/sprime, last accessed August 17, 2022), and SPrime pipeline ([Zhou and Browning 2021]; https://github.com/YingZhou001/sprimepipeline, last accessed August 17, 2022). Demographic models in Demes YAML format (Gower et al. 2022) can be found in https://github.com/admixVIE/sstar-analysis/tree/main/config/simulation/models (last accessed August 17, 2022).
